# Tau pathology in triple knock‐in mouse model of Alzheimer's Disease expressing APOE3 and APOE4

**DOI:** 10.1002/alz70855_101626

**Published:** 2025-12-23

**Authors:** Jamie L Fournier, Aya Arrar, Madison R Longmuir, Taylor W. Schmitz, Lisa M Saksida, Timothy J Bussey, Vania F Prado, Marco AM Prado

**Affiliations:** ^1^ University of Western Ontario, London, ON, Canada; ^2^ Robarts Research Institute, London, ON, Canada; ^3^ Schulich School of Medicine & Dentistry, London, ON, Canada; ^4^ Schulich Medicine and Dentistry, London, ON, Canada; ^5^ Schulich School of Medicine and Dentistry, London, ON, Canada

## Abstract

**Background:**

Tau is a microtubule stabilizing protein that becomes dysfunctional during the course of Alzheimer's Disease. Previous research has shown that individually amyloid and APOE4 have the potential to increase tau phosphorylation and worsen tau pathology in tau mutant models of tauopathy. However, whether both amyloid and APOE4 can further contribute to tau dysfunction in mouse models that more faithfully reflect the levels of tau in humans is unknown. We developed mouse models with a combination of incorporated humanized knock‐in variants of hMAPT, App^NL^ and App^NL‐F^ and APOE 3 or APOE 4 genotypes.

**Method:**

Biochemical and imaging techniques including immunofluorescence microscopy and Western Blots were performed for phosphorylated tau at the Serine 202/Threonine 205 sites (AT8) and total tau. Visuospatial learning and memory were assessed using the Paired Associates Learning (PAL) task, an automated touchscreen task.

**Result:**

Our preliminary results indicated that tau protein phosphorylated at the Serine 202/Threonine 205 phosphorylation site is detected in in both male and female mice in a genotype dependent manner. Aging, expression of App ^NL‐F^ and APOE4 increased tau phosphorylation. Insoluble total tau was found predominantly in aged mice (18 months) and was worse in APOE4 expressing individuals. All the antibodies used detected no signal in tissue from tau knockout mice confirming the results are a consequence of changes in tau. Preliminary results indicate that 12‐month old App ^NL^ and App ^NL‐F^ mice (ApoE3 and ApoE4) were able to learn the PAL task to 70% accuracy. There were no genotype‐dependent differences in performance.

**Conclusion:**

The results of this project demonstrate that tau pathology markers appear to increase as a function of age, APOE4 and the ability to accumulate insoluble Abeta and plaques^.^ These results agree with the notion that amyloid and APOE4 can increase tau markers even in less aggressive animal models without tau overexpression or mutations. Our findings also suggest that there are no genotype‐dependent differences in learning and memory in the PAL task at early ages, despite these mice presenting other cognitive deficits at that stage.

